# Brazilian green propolis promotes TNFR2 expression on regulatory T cells

**DOI:** 10.1002/fsn3.2281

**Published:** 2021-04-07

**Authors:** Norihisa Mikami, Hiroko Tani, Ryoji Kawakami, Atsushi Sugimoto, Shimon Sakaguchi, Tomoki Ikuta

**Affiliations:** ^1^ Department of Experimental Immunology Immunology Frontier Research Center Osaka University Suita Japan; ^2^ Institute for Bee Products and Health Science Yamada Bee Company, Inc. Okayama Japan

**Keywords:** artepillin C, Brazilian green propolis, regulatory T cells, TNFR2

## Abstract

FoxP3^+^ regulatory T cells (Tregs) are needed to suppress inflammatory diseases and maintain immune homeostasis. The suppressive function of Tregs can be used to control autoimmune or inflammatory diseases; therefore, it is well studied how Tregs can be artificially up‐ or downregulated in vitro and in vivo, by using antibodies, chemical compounds, foods, and natural resources. Propolis is a famous functional food that has an anti‐inflammatory effect. However, the influences of propolis on Treg function have not been fully evaluated so far. Here, we demonstrated that Brazilian green propolis increases TNFR2 expression in Tregs via the IRF4/cMyc axis, and artepillin C was a major effective component of propolis on Tregs. These results indicate that propolis and artepillin C have the potential as Treg activators via TNFR2 expression and may be useful for the prevention and/or therapy of autoimmune or inflammatory diseases.

## Graphical abstract

Bee product of propolis is a famous functional food that has an anti‐inflammatory effect. We showed Brazilian green propolis increases TNFR2 expression through the IRF4/cMyc axis in Tregs, and artepillin C was a major effective component of propolis on Tregs. These results indicate that propolis and artepillin C have the potential as a Treg activator via TNFR2 expression and may be useful for the prevention and/or therapy of autoimmune or inflammatory diseases.

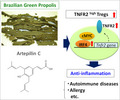

## 1 INTRODUCTION

FoxP3^+^ regulatory T cells (Tregs) suppress a variety of immune responses and help maintain immune self‐tolerance and homeostasis (Sakaguchi et al., 2008, 2020). These natural occurring Tregs (nTregs) originate from the thymus. Recently, there is accumulating evidence to suggest that FoxP3^+^ Tregs are adaptive in the periphery, are able to proliferate clonally following in vivo antigenic stimulation, and consequently exert antigen‐specific immune suppression (Shevach & Thornton, 2014). It has also been well established that in vitro stimulation in the presence of TGF‐β is able to induce FoxP3 expression in conventional T (Tconv) cells (Chen et al., 2003), while this in vitro generation of TGF‐β‐dependent inducible Tregs (iTregs) is unable to develop functionally stable iTregs due to their failure to acquire stable Treg‐specific epigenomic changes in *Foxp3* and other Treg‐DR genes (Floess et al., 2007; Ohkura et al., 2012; Polansky et al., 2008; Shevach & Thornton, 2014).

These findings have led us to recently develop the method of manipulating several Treg subpopulations and treating immunological disorders. In clinical trials, Treg‐based immune therapy is already being examined and its efficacy has been well studied (Sakaguchi et al., 2020). In autoimmune diseases, the in vivo expansion of Tregs appears to be a good therapeutic way, and several studies have provided effective treatments, such as the administration of IL‐2 (Sakaguchi et al., 2020). Therefore, in order to prevent autoimmune or inflammatory diseases, it is important to find compounds or natural resources that modulate Treg function.

Propolis is a natural product collected by honey bees from various resinous secretions of plants such as gums, resins, and also from the leaf buds of some types of plant. Propolis has traditionally been used as a folk medicine and is used for various purposes including antimicrobial, anti‐oxidant, anti‐inflammatory, and antitumor properties. More than hundreds of constituents have been identified in propolis. As the major ingredients of propolis are derived from plants, the chemical composition of propolis depends to a large extent on its geographical and botanical origin, the season, and the method of extraction (Anjum et al., 2019). Brazilian green propolis, which is produced in the southeast of Brazil, is rich in organic substances mainly from *Baccharis dracunculifolia* (Park et al., 2004). Cinnamic acid derivatives, flavonoids, and caffeoylquinic acid derivatives are mainly contained in the ethanolic extracts of Brazilian green propolis (Tani et al., 2019). In particular, artepillin C has the highest content in Brazilian green propolis (5.0%–11.0%; Matsuda & Almeida‐Muradian, 2008).

The anti‐inflammatory activity of Brazilian green propolis has been reported for seasonal allergic rhinitis (Takeuchi et al., 2009a, 2009b), chronic periodontitis (Nakao et al., 2020), gastric ulcers (Costa et al., 2020), colitis (Shimizu & Suzuki, 2019), hepatocellular inflammation (Tsuchiya et al., 2018), and pancreatitis (Al‐Hariri et al., 2019). Furthermore, clinical trials have shown a reduction in systemic inflammation (Zhu et al., 2018). The anti‐inflammatory mechanism of action of propolis has been reported and consists of Th1/Th2 balance (Pagliarone et al., 2009), antihistamine (Shaha et al., 2018; Shinmei et al., 2004), antileukotriene (Tani et al., 2010), and modulation of macrophage functions (Szliszka et al., 2013). However, the effects of propolis on Tregs have not yet been fully studied.

In the present manuscript, we investigated the effects of Brazilian green propolis on Tregs in vitro and in vivo, as well as the molecular key of propolis function using RNA‐sequencing analysis, and the major active compound of propolis. These findings provided a new valence of propolis for regulating Treg function and immune responses.

## 2 MATERIALS AND METHODS

### 2.1 Mice

Male C57BL/6 and BALB/c mice (6–8 weeks old) were purchased from SLC or CLEA. Male Foxp3‐eGFP (eFox) reporter mice were previously described (Akamatsu et al., 2019). All procedures were performed in accordance with the Guide of the National Institutes of Health for the Care and Use of Laboratory Animals and approved by the Animal Research Committee of the Osaka University (R1‐03‐0). All efforts were made to minimize suffering.

### 2.2 Reagents

Recombinant human IL‐2 and human TGF‐β1 were purchased from Shionogi & Co., Ltd. and R&D systems, Inc., respectively. 10058‐F4 (cMyc inhibitor) was purchased from Abcam.

Anti‐mouse CD4 (RM4‐5), anti‐mouse CD25 (PC61), anti‐mouse CD44 (IM7), anti‐mouse CD62L (MEL‐14), and anti‐mouse GITR (DTA‐1) were purchased from BD Biosciences. Anti‐mouse CD38 (90), anti‐mouse CTLA4 (UC10‐4B9), anti‐mouse ICOS (C398.4A), and anti‐mouse FoxP3 (FJK‐16s) were purchased from eBioscience. Anti‐mouse‐TNFR2 (TR75‐89) and anti‐mouse IRF4 (IRF4.3E4) were purchased from BioLegend.

The Brazilian green propolis ethanol extract was obtained from Yamada Bee Co., Inc. (Lot. 140605). It is standardized to contain 8.0% artepillin C and 0.14% culifolin. The HPLC‐DAD chromatogram of the Brazilian green propolis ethanol extract has been previously reported (Tani et al., 2019). Artepillin C, caffeic acid, and kaempferide were purchased from Wako Co.. Chlorogenic acid and *p*‐coumaric acid were obtained from Sigma. Betuletol was purchased from Nacalai Tesque. Drupanin, baccharin, and culifolin were prepared by our previously reported method (Tani et al., 2010). Capillartemisin A, 2,2‐dimethylchromen‐6‐(*E*)‐propenoic acid, dihydrokaempferide, and drupanal were prepared by our previously reported method (Tani et al., 2019). The purity of these reference compounds was determined to be greater than 98% by normalizing the peak area detected by HPLC analysis (275 nm) and ^1^H NMR spectrum.

### 2.3 Cell isolation and flow cytometry analysis

T‐cell sorting was performed as previously described (Akamatsu et al., 2019; Mikami et al., 2020; Sugimoto et al., 2017). In order to prepare the cells for culture experiments, FACSAriaII (BD) was used to collect a specific population. Specific cell populations were defined as follows: naive T cells, CD4^+^GFP^−^CD44^low^CD62L^high^; nTreg cells, CD4^+^GFP^+^; and conventional T (Tconv) cells, CD4^+^GFP^−^. When intracellular staining was required, cell fixation and permeabilization were performed using the Foxp3/Transcription Factor Staining Buffer Set (eBioscence) or BD Pharmingen Transcription Factor Buffer Set (BD) according to the manufacturer's instructions.

### 2.4 Stimulation of nTreg and induction of iTreg cells

For cell culture, we used RPMI1640 supplemented with 10% FCS (v/v), 60 µg/ml penicillin G, 100 µg/ml streptomycin, and 0.1 mM 2‐mercaptoethanol. To stimulate nTreg cells, 2 × 10^5^ sorted Treg cells were stimulated with Dynabeads mouse T‐cell activator in the presence of 100 U/ml human IL‐2. To induce iTreg cells, 2 × 10^5^ sorted Tconv or Tnaive cells were stimulated with Dynabeads mouse T‐cell activator in the presence of 50 U/mL of human IL‐2 and 5 ng/ml of human TGF‐β1, in 96‐well flat‐bottom plates (Thermo Scientific, #167008) for 3 days. Propolis and its components were treated at the same time with Dynabeads mouse T‐cell activator to analyze its effect on nTreg or iTreg cells.

### 2.5 Propolis treatment

Mice were treated with 50 mg/kg of propolis in 0.5% methyl cellulose by gavage for one week, every day. ICOS and TNFR2 expression on nTregs was determined by FCM analysis. For the dose setting, we have referred to previous reports of propolis for the allergic rhinitis (Shaha et al., 2018).

### 2.6 RNA‐sequencing analysis

The RNA‐sequencing method has been previously described (Akamatsu et al., 2019). Briefly, after in vitro stimulation, Tregs were lysed in RLT buffer (Qiagen) containing 1% 2‐mercaptoethanol, followed by reverse RNA transcription with SMART‐seq v4 Ultra Low Input RNA Kit for Sequencing (Clontech). After cDNA fragmentation by the KAPA Frag kit (KAPA), the sequence libraries were prepared using the KAPA Library IonTorrent (KAPA) preparation kit following the manufacturer's protocol. The cDNA library sequence was performed by IonS5 (Thermo Scientific).

The acquired sequencing results were mapped to the mouse reference genome (mm9; UCSC) using tophat2, and the unmapped sequences were mapped by bowtie2. The combined mapped readings were used to calculate normalized FPKM values using Cuffnorm of Cufflinks package (version 2.2.1, Trapnell Lab) in the default settings.

### 2.7 Statistics

Values were expressed as mean ± *SD*. Statistical significance was assessed by the paired or unpaired Student's *t* test (two groups), nonrepeated measures analysis of variation (ANOVA) followed by the Bonferroni test (vs. control), Dunnett test, or Student–Newman–Keuls test (multiple comparisons). A probability of less than 5% (*p* < .05) was considered statistically significant.

## 3 RESULTS

### 3.1 The effects of Brazilian green propolis on Tregs

**FIGURE 1:**
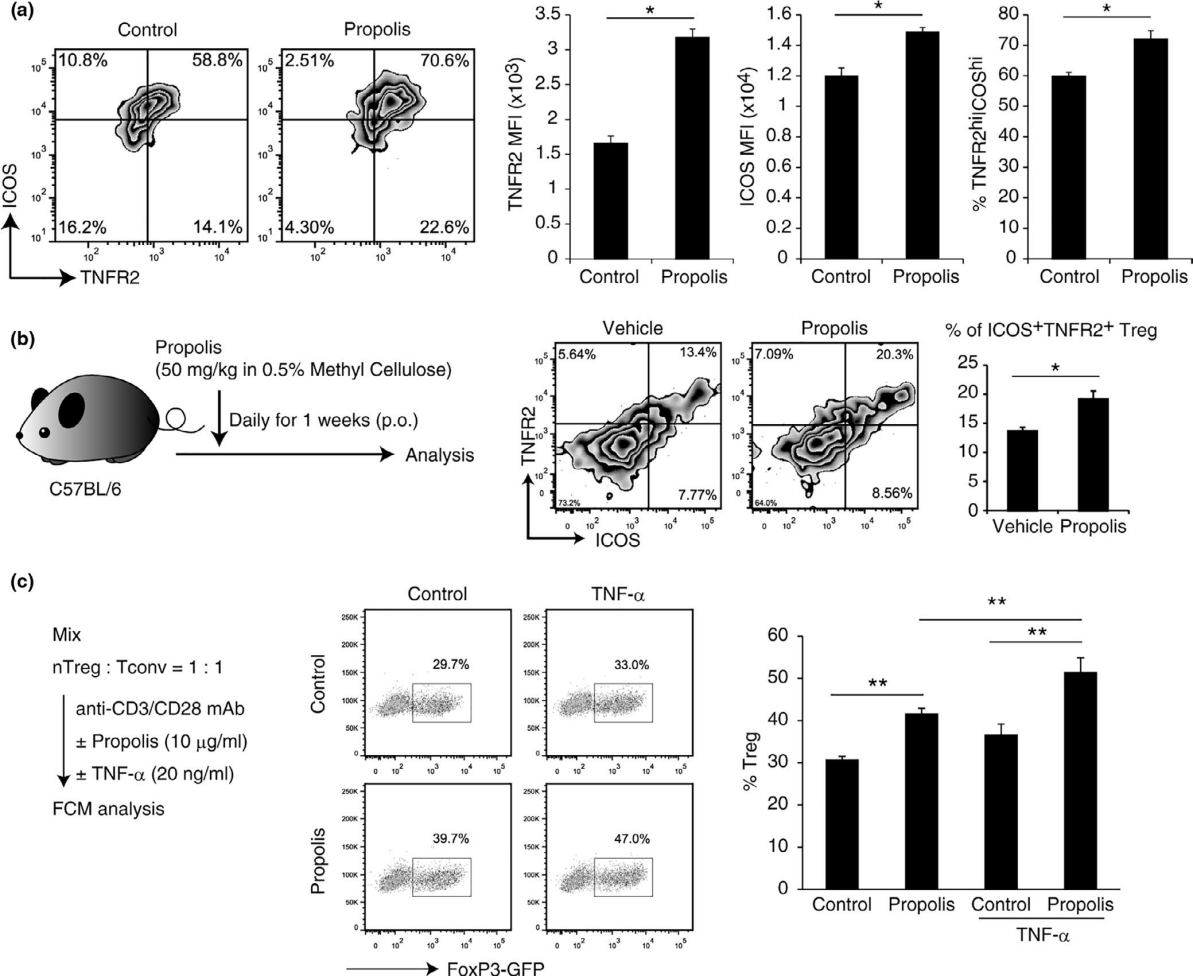
Propolis induces TNFR2 expression and Treg expansion. (a) The effect of propolis on nTreg was determined by FCM analysis. Cells were stimulated for 72 hr with propolis (10 μg/ml) and anti‐CD3/CD28 Dynabeads in the presence of 100 U/ml IL‐2. (b) Mice were administrated 50 mg/kg propolis (p.o.) for one week. ICOS and TNFR2 expression on nTregs were determined by FCM analysis. (c) The expansion of Treg was determined by FCM analysis. The Treg and Tconv mixture (1:1) was stimulated for 72h with propolis (10 μg/ml) and anti‐CD3/CD28 Dynabeads in the presence of 100 U/ml IL‐2. Vertical bars represent means ± *SD*; *n* = 3, *p < .05, **p < .01 (Student's *t* test or SNK method)

We first examined the effect of propolis on both iTregs and nTregs in vitro. Because high dose of propolis affects the activation and/or survival (Figure S1) of T cells, we used less than 30 μg/ml propolis for experiments to avoid the subeffect of propolis such as cell death. The propolis did not affect the expression of Treg‐signature genes such as FoxP3, GITR, CD25, CTLA4, or Helios in iTregs (Figure S2). The expression of these molecules was never altered in nTregs, while the TNFR2 expression in nTregs was specifically upregulated by propolis (Figure 1a; Figure S2).

TNFR2 is expressed in activated Tregs and contributes to their expansion in the inflammatory or tumor tissue environment when TNF‐α is involved (Chen et al., 2007, 2008; Okubo et al., 2013). In fact, staining in combination with ICOS, another Treg‐specific active molecule (Chen et al., 2018), clearly showed that propolis increased the percentage of TNFR2^high^ICOS^high^ Treg population both in vitro and in vivo (Figure 1a,b), suggesting that propolis can promote TNFR2 expression in activated Tregs and enhances its proliferative or activating potential.

In order to achieve Tregs enrichment, we mixed nTreg and Tconv cells in a 1:1 ratio and stimulated them with anti‐CD3/CD28 mAb in the presence or absence of propolis and/or TNF‐α. As the result, propolis treatment succeeded in enriching Tregs and the effect was increased by combined treatment with TNF‐α (Figure 1c). This indicates that propolis promotes TNFR2 expression and, consequently, increases reactivity to TNF‐α.

### 3.2 The mechanism of TNFR2 induction by Brazilian green propolis

**FIGURE 2:**
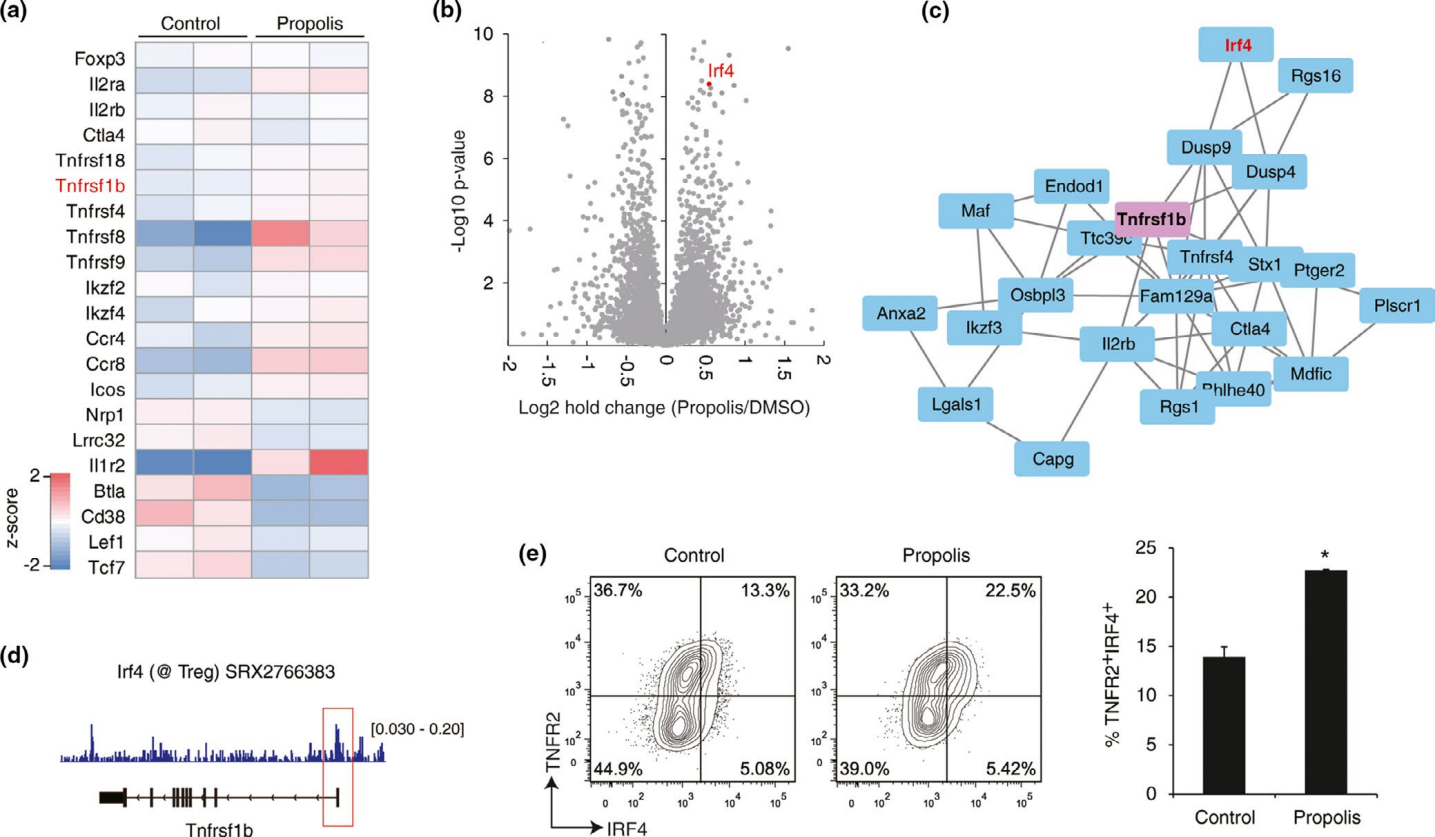
TNFR2 regulation by propolis and IRF4. (a, b) Gene expression of nTreg cells was analyzed by RNA‐sequencing analysis. The nTreg cells were stimulated for 72h with or without propolis (10μg/ml). The heat map (a) and volcano plot (b) are shown. Data were obtained from two independent experiments. (c) The correlation network of TNFR2 gene expression in nTreg cells was obtained from Immuno‐Navigator (https://genomics.virus.kyoto‐u.ac.jp/immuno‐navigator/). (d) IRF4 binding to the promoter region of the TNFR2 gene was obtained from the indicated public data (GSM2590030). (e) TNFR2/IRF4 expression in nTreg cells was determined by FCM analysis in the presence or absence of 10μg/ml propolis. The data were represented by two independent experiments. Vertical bars represent means ± *SD*; *n* = 3, *p < .05 (Student's *t* test)

In order to evaluate the global gene expression pattern and the functional mechanism of propolis, RNA‐sequencing analysis was carried out by using nTregs. Some of the Treg‐signature genes, such as *Tnfrsf*s including *Tnfrsf1b* (encodes TNFR2), *Ccr4*, *Ccr8*, and *Icos*, were upregulated by propolis (Figure 2a). On the other hand, *Cd38*, *Lef1*, and *Tcf7* were downregulated (Figure 2a). These results suggested that propolis somehow modulates Treg function and its effect on TNFR2 expression is due to transcriptional regulation.

Next, we approached the TNFR2 induction mechanism. According to the RNA‐sequencing analysis, the interferon regulatory factor 4 (IRF4) was found as a transcription factor upregulated by propolis (Figure 2b). Furthermore, the correlation between TNFR2 and IRF4 was observed in the public Treg transcriptome data (Figure 2c, obtained from Immuno‐Navigator (Vandenbon et al., 2016)). Public ChIP‐sequencing data also indicated that IRF4 has possible to bind directly to the transcription start site of TNFR2 gene in Tregs (Figure 2d). Flow cytometric analysis demonstrated the upregulation of TNFR2^+^IRF4^+^ fraction by propolis (Figure 2e). As a result, the staining pattern showed that TNFR2‐expressing Tregs had a higher IRF4 expression than the negative‐TNFR2 population. Therefore, these data strongly suggested that TNFR2 expression would be enhanced by the IRF4 transcription factor and would play a key role in the upregulation of propolis‐mediated TNFR2.

**FIGURE 3:**
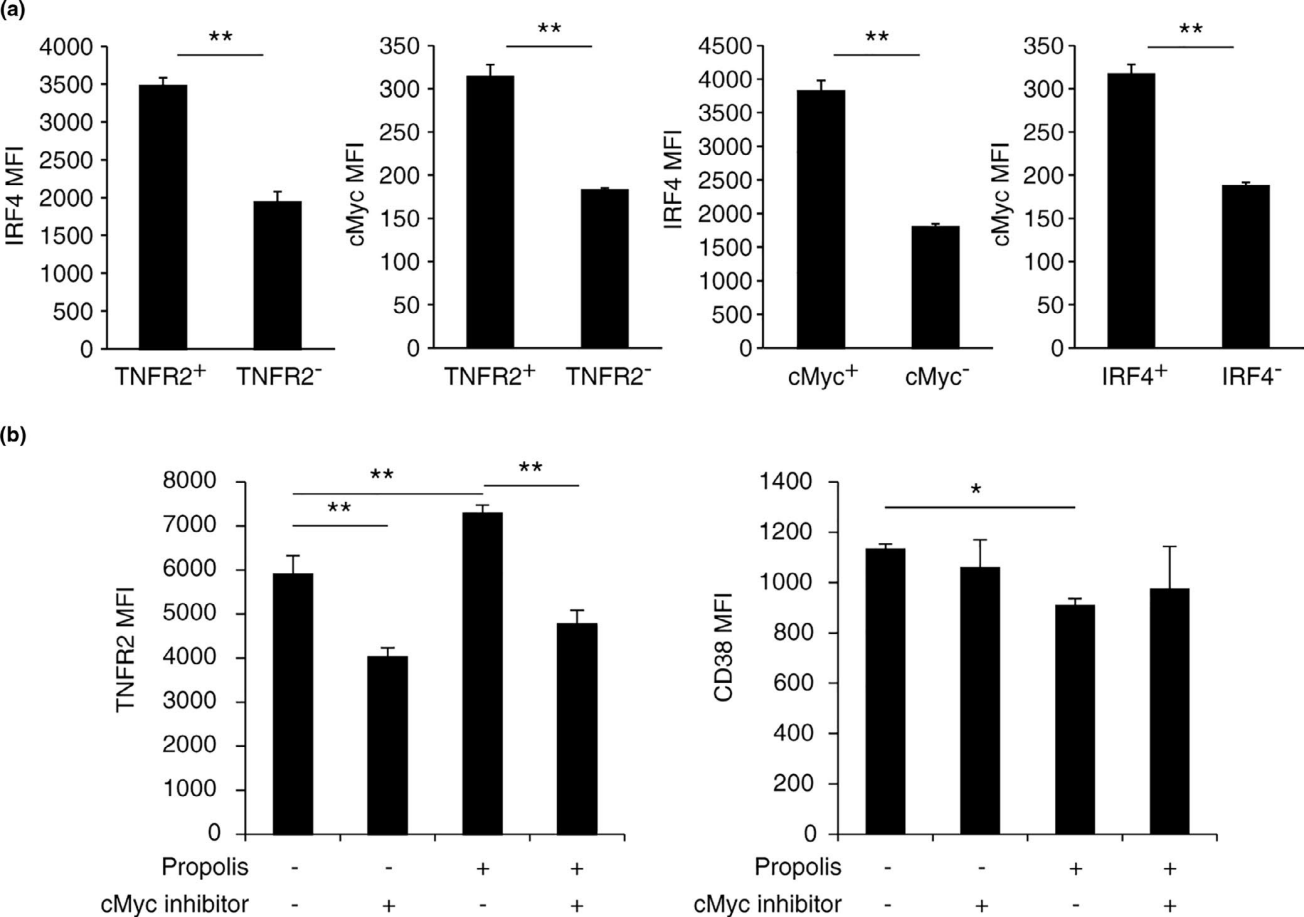
Effect of propolis and cMyc inhibitor on TNFR2 and CD38. (a) Expression of IRF4 and cMyc in stimulated nTreg cells were determined by FCM analysis. (b) TNFR2 and CD38 expression was determined by FCM analysis. nTreg cells were stimulated for 72 hr with anti‐CD3/CD28 Dynabeads and 100 U/ml IL‐2 in the presence or absence of 50 μM 10058‐F4 and/or 10 μg/ml propolis. Vertical bars represent means ± *SD*; *n* = 3, *p < .05, **p < .01 (Student's *t* test or SNK method)

In order to approach the direct interaction between propolis and TNFR2, we focused on the transcription factor cMyc as a synergistic collaborator of IRF4 (Gopalakrishnan et al., 2016; Weilemann et al., 2015). In particular, there were positive correlations among TNFR2, IRF4, and cMyc in activated nTregs (Figure 3a). Because there is no viable IRF4 inhibitor, we used the cMyc inhibitor to assess the in vitro function of propolis. Remarkably, TNFR2 expression that is increased with propolis was blocked by the cMyc inhibitor (Figure 3b left). On the other hand, CD38 expression, which was shown as a downregulated gene in Figure 2a, was significantly reduced by propolis (Figure 3b right). However, the CD38 expression and cMyc inhibitor were not significantly different in the presence or absence of propolis treatment in nTreg cells, respectively (Figure 3b right). Although CD38 regulation of propolis has not been proven, our findings suggested that TNFR2 was regulated by the IRF4/cMyc axis as a major propolis signaling pathway.

### 3.3 The effect of Brazilian green propolis components on Tregs in vitro

In order to approach the critical component of propolis for TNFR2 regulation, we examined the TNFR2 expression on Tregs treated with propolis and its 13 components (Figure S3). These results showed that propolis, artepillin C, drupanal, and dihydrokaempferide had the same tendency of upregulating TNFR2 and downregulating CD38 (Figure S3). Given that the average content of artepillin C was almost one‐tenth (9.81%) of propolis (Tani et al., 2019), and the effective concentration of artepillin C (1 μg/ml) was one‐tenth lower than that of propolis (10 μg/ml), artepillin C may be the main functional component of propolis.

**FIGURE 4:**
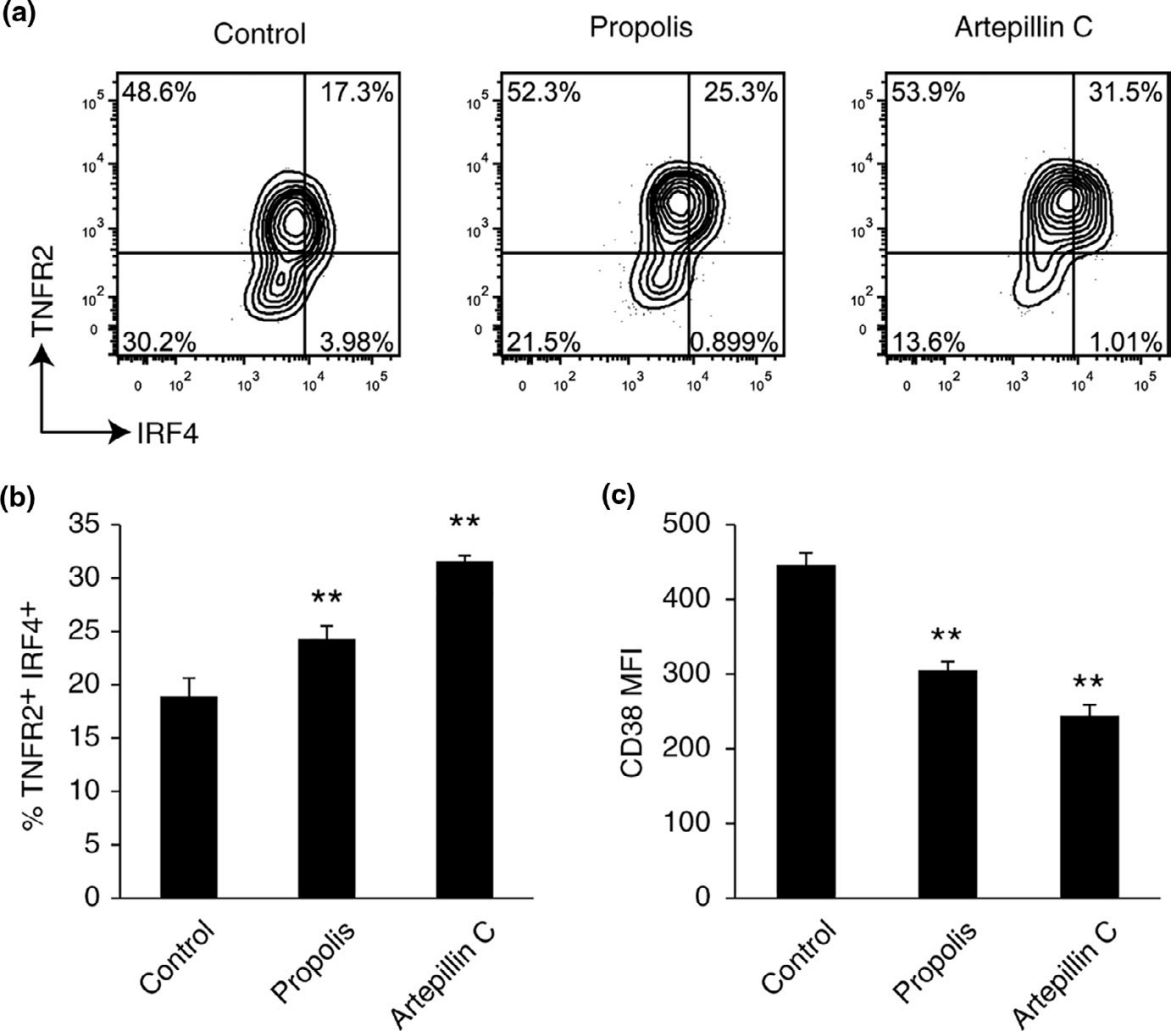
Effect of Brazilian green propolis components on Tregs in vitro (a, b) TNFR2/IRF4 expression in nTreg cells was determined by FCM analysis in the presence of 10 μg/ml propolis or 1 μg/ml artepillin C. (c) CD38 expression in nTreg cells was determined by FCM analysis. Vertical bars represent means ± *SD*; *n* = 3, *p < .05, **p < .01 (Bonferroni test, vs. control group)

To confirm it, we strictly investigated the TNFR2, IRF4, and CD38 expression in propolis and artepillin C—treated Tregs. Flow cytometric analysis demonstrated that propolis and artepillin C induced high expression of TNFR2 and IRF4 on Tregs (Figure 4a,b). In addition, downregulation of CD38 by artepillin C was observed and is consistent with the effect of propolis (Figure 4c). These results revealed that artepillin C was a major effective component of propolis on Tregs.

## 4 DISCUSSION

In this study, we provided the first report that Brazilian green propolis increases TNFR2 expression through the IRF4/cMyc axis in Tregs, and artepillin C was a major effective component of propolis on Tregs.

Tregs play important roles in allergy, autoimmunity, infection, and tumor immunity (Sakaguchi et al., 2008, 2020). Effect of propolis on Tregs has been reported that the frequency of Tregs was increased by propolis in mice model of allergic airway inflammation (Piñeros et al., 2020). On the other hand, Cheung et al. (2011) reported that Foxp3 expression did not change in Tregs treated with propolis, and the conclusion was that propolis had no effect on Treg induction (Cheung et al., 2011). Our results showed that propolis never affected FoxP3 expression in iTreg and nTreg (Figure S2), being consistent with their studies. In the present study, focused on TNFR2, a new function of propolis on Tregs was revealed. Recently, TNFR2, a TNF receptor, is reported as a novel Treg‐specific functional receptor and contributes to the suppressive function of Tregs (Chen et al., 2007, 2008; Okubo et al., 2013). TNF has two receptors, TNFR1 and TNFR2. TNFR1 has ubiquitous cell expression, while TNFR2 is restricted to the subpopulations of T cells, endothelial cells, and neurons (Okubo et al., 2013). Although TNF is a major proinflammatory cytokine, TNF also has an immunosuppressive feedback effect by Tregs. TNFR2^+^Tregs was the maximum suppressive subset of Tregs (Chen et al., 2008). In addition, the selective TNFR2 agonist allowed the expansion of Tregs which protected against immunological rejection of hematopoietic stem cell transplantation. Therefore, some recent Treg‐based therapies for inflammatory diseases have focused on Treg expansion in vivo by administration of the TNFR2 agonist (Chopra et al., 2016). We have also shown that propolis enhances TNF‐mediated Treg expansion in vivo (Figure 1b), so treatment with propolis or artepillin C as TNFR2 agonist is expected to be a good anti‐inflammatory therapy.

IRF4^+^ Treg has superior suppressive activity (Alvisi et al., 2020). RNA‐sequencing analysis revealed the transcription factor IRF4, which was upregulated by propolis and had the potential to bind the promoter region of TNFR2. Moreover, TNFR2 expression on Tregs is correlated with IRF4 and cMyc expression and indeed reduced by the cMyc inhibitor (Figure 3b). Therefore, propolis has the potential to adjust to Tregs through the IRF4/cMyc axis. Notably, propolis did not induce TNFR2 expression in iTregs, which was induced by TGFβ1 (Figure S2). Because TGFβ1 inhibits cMyc expression in T cells (Genestier et al., 1999), TGFβ1 could inhibit TNFR2 expression by downregulating cMyc on iTregs despite propolis treatment.

Furthermore, we found that CD38 was suppressed by propolis (Figures 2a and 3b). CD38 is expressed on hematopoietic cells including Tregs and is a transmembrane glycoprotein that acts as an enzyme and receptor. A high CD38 expression on Tregs showed superior suppressive activity (Patton et al., 2011). The combination with TNFR2^high^ and CD38^low^ on Tregs may have a role on TNF‐dependent Tregs, and physiological activity remains to be investigated.

In the present study, artepillin C, a cinnamic acid derivative, was a major effective component of propolis on Tregs. According to the structure–activity relationships, two isoprenyl groups of artepillin C will be necessary to upregulate TNFR2 expression on Tregs.

The biological activity of artepillin C has been reported to reduce the inflammatory response by inhibiting NFκB on macrophages (Szliszka et al., 2013), to increase cytotoxic activity on natural killer cells (Takeda et al., 2018) to inhibit the release of cys‐leukotrienes from the HL‐60 leukemia cell line (Tani et al., 2010). Although the relationships between IRF4/cMyc and Tregs have been reported (Koizumi et al., 2018; Saravia et al., 2020), the direct target of artepillin C and the stream of the IRF4/cMyc axis are still unclear. A target of artepillin C, TRPA1, is known to be expressed on T cells (Bertin et al., 2017; Hata et al., 2012), so that the related signaling pathway may be involved in effect. Further investigation is needed to clarify the propolis signaling cascade, while propolis is the first TNFR2‐inducing substance and its effect on Tregs would be one of the mechanisms explaining the anti‐inflammatory efficacy, such as improving seasonal allergic rhinitis (Takeuchi et al., 2009a, 2009b) and reducing systemic inflammation (Zhu et al., 2018).

In conclusion, Brazilian green propolis induced the highest expression of TNFR2 in Tregs and supported their expansion and activation. Propolis and its components, especially artepillin C, have the potential as a Tregs activator through TNFR2 expression. Furthermore, these findings would promote the development of prevention and/or therapy for inflammation or immune diseases, focusing on the Treg expansion by the TNFR2 agonist.

## CONFLICT OF INTEREST

T.I. and H.T. are employees of Yamada Bee Company Inc. N.M. received a research grant from Yamada Bee Company Inc. Other authors have no conflicts of interest.

## AUTHOR CONTRIBUTION

**Norihisa Mikami:** Conceptualization (equal); Data curation (lead); Formal analysis (lead); Funding acquisition (lead); Investigation (equal); Project administration (equal); Writing‐original draft (lead). **Hiroko Tani:** Resources (lead). **Ryoji Kawakami:** Investigation (equal); Methodology (equal). **Atsushi Sugimoto:** Investigation (equal); Methodology (equal). **Shimon Sakaguchi:** Supervision (lead). **Tomoki Ikuta:** Conceptualization (equal); Project administration (equal); Writing‐review & editing (lead).

## Supporting information

Fig S1

Fig S2

Fig S3

## Data Availability

Next‐generation sequencing data were stored in the DDBJ Sequence Read Archive with accession number DRA010492.
