# Supplementary figures and images for: Brazilian green propolis promotes TNFR2 expression on regulatory T cells
Source: Food Sci Nutr. 2021 Apr 7;9(6):3200–8. doi: 10.1002/fsn3.2281 (PMC8194755; doi:10.1002/fsn3.2281)

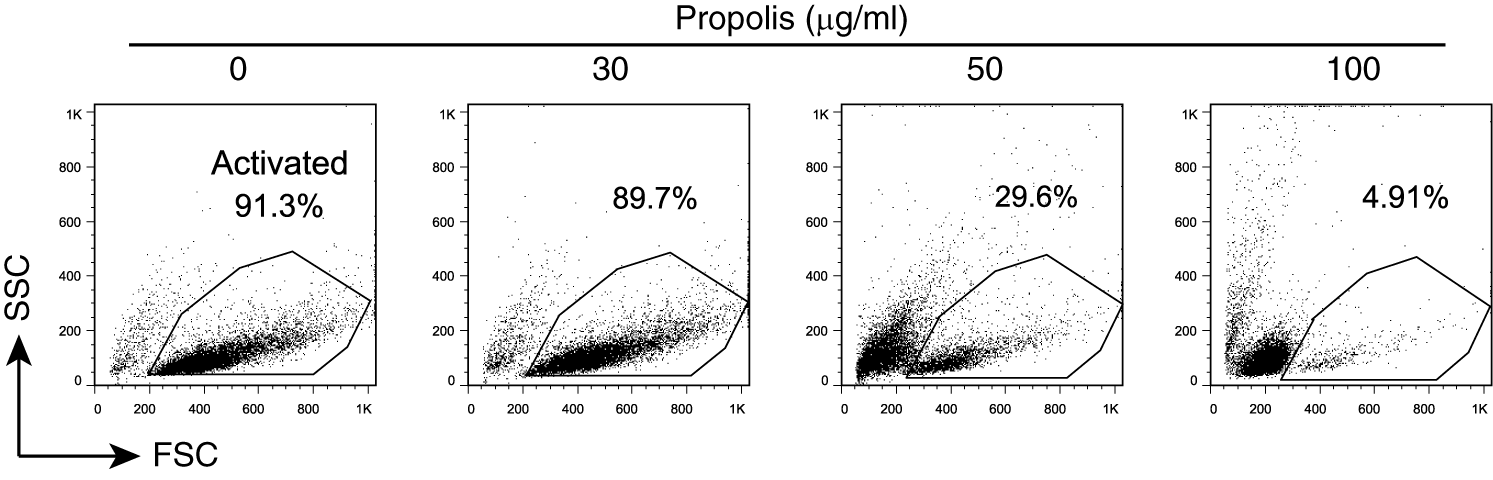

Supplement: Supplementary file 1 — Fig S1 [file FSN3-9-3200-s001.tif]

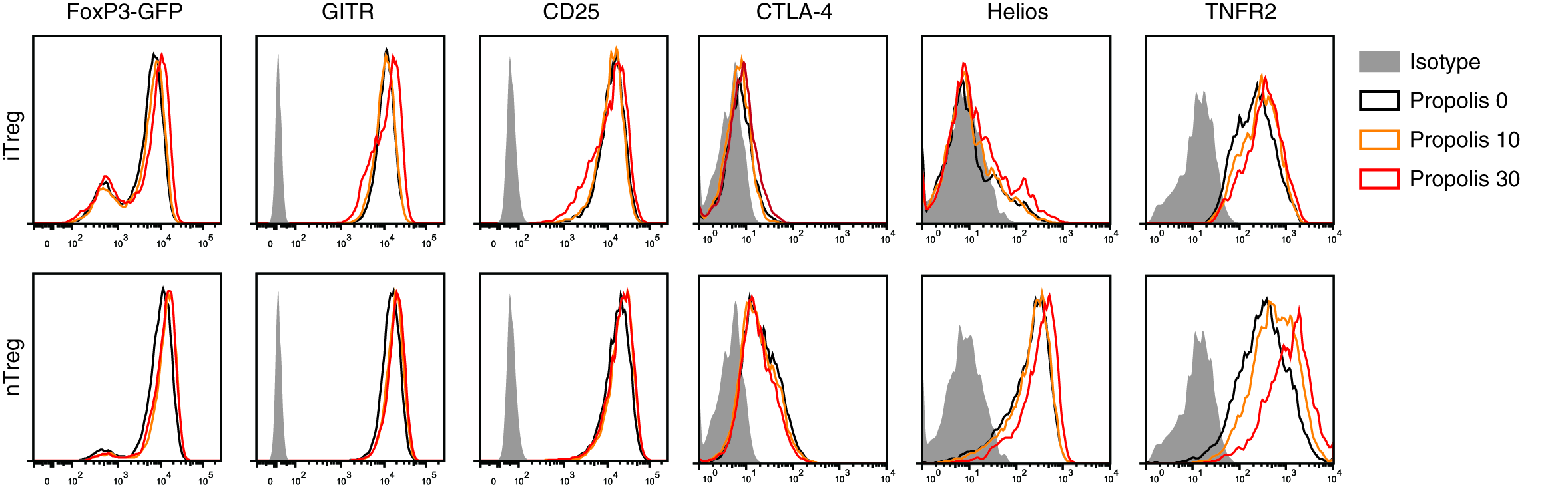

Supplement: Supplementary file 2 — Fig S2 [file FSN3-9-3200-s003.tif]

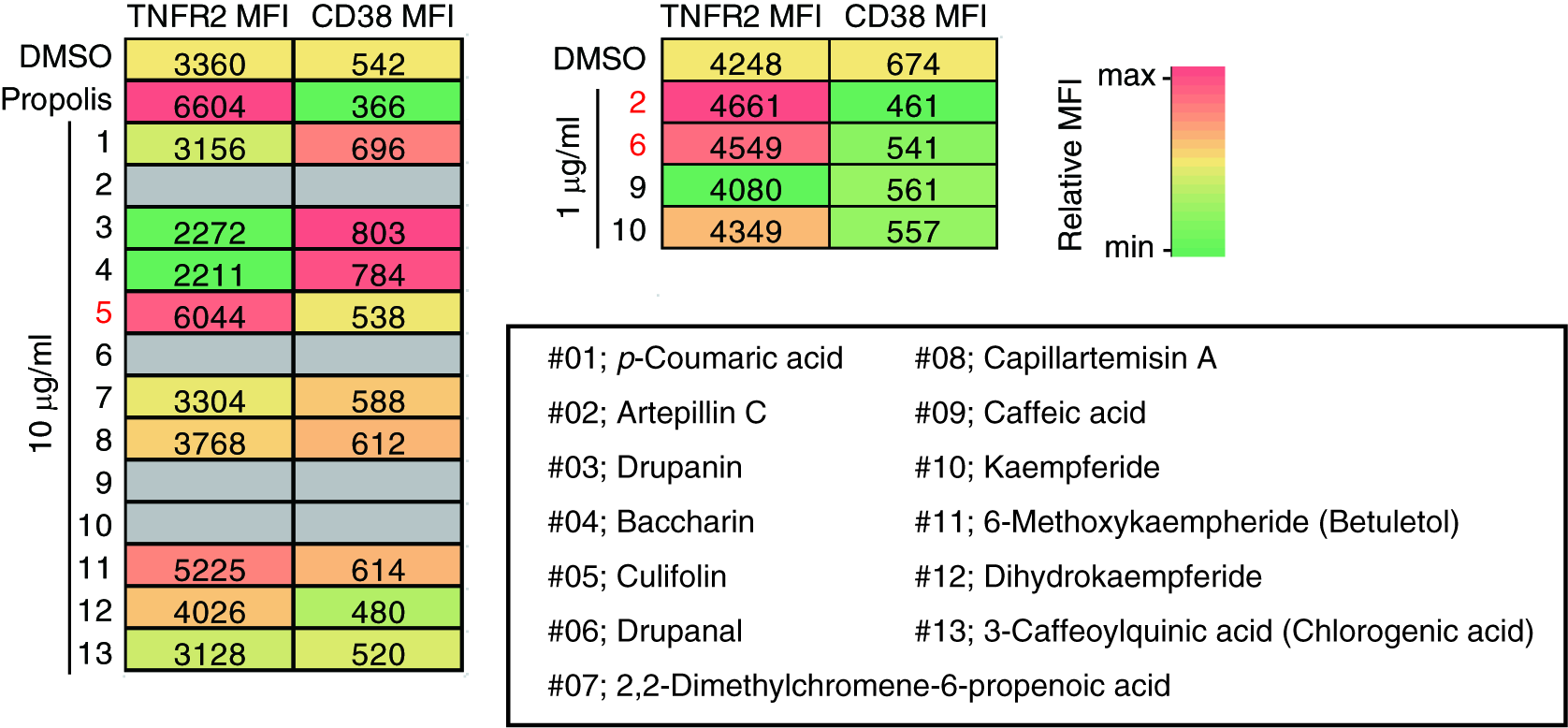

Supplement: Supplementary file 3 — Fig S3 [file FSN3-9-3200-s002.tif]
